# Diet supplemented with fermented okara improved growth performance, meat quality, and amino acid profiles in growing pigs

**DOI:** 10.1002/fsn3.1857

**Published:** 2020-09-07

**Authors:** Zhimei Tian, Dun Deng, Yiyan Cui, Weidong Chen, Miao Yu, Xianyong Ma

**Affiliations:** ^1^ State Key Laboratory of Livestock and Poultry Breeding Key Laboratory of Animal Nutrition and Feed Science in South China Ministry of Agriculture and Rural Affairs Guangdong Key Laboratory of Animal Breeding and Nutrition Guangdong Engineering Technology Research Center of animal Meat quality and Safety Control and Evaluation Institute of Animal Science Guangdong Academy of Agricultural Sciences Guangzhou China

**Keywords:** amino acid profile, fermented okara, growing pigs, growth performance, meat quality

## Abstract

This study aimed to assess the efficacy of fermented okara on performance and meat quality, and to explore the feasibility of its partial substitution for corn‐soybean meal in pig production. A total of 48 pigs (Duroc × Landrace × Yorkshire) with an average body weight of 58.60 ± 0.65 kg were randomly assigned to 2 groups, Control group and Fermented okara (FO) group. There were 8 replicate pens each with 3 pigs per treatment. Control pigs were fed a corn‐soybean meal basal diet, treatment pigs were fed a basal diet supplemented with FO throughout the 55‐d experimental period. Results showed that fermentation of okara using probiotics increased its microporous structure, polysaccharides, lactic acid, and free amino acids (FAA) by 46.06%, 150%, and 66.45% compared with unfermented okara, respectively (*p* < .05). The diet supplemented with FO significantly improved average daily gain (ADG) by 8.70% (*p* < .01), but decreased the feed gain ratio (F/G) by 5.56% of growing pigs compared to the control diet (*p* < .05). Furthermore, dietary FO improve meat color, FAA, and the activity of total superoxide dismutase (T‐SOD) and glutathione peroxidase (GSH‐PX) in the serum and muscles (*p* < .05). Collectively, probiotics‐fermented okara improved growth performance, meat quality and antioxidant capacity, and it can be used to substitute partial corn‐soybean meal in pig industry.

## Graphical abstract

Fermentation of okara using probiotics increased its microporous structure, polysaccharides, lactic acid, and free amino acids. Diet supplemented with fermented okara improved growth performance, meat quality and antioxidant capacity, and it can be used to substitute partial corn‐soybean meal in pig industry.

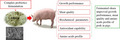

## 1 INTRODUCTION

Along with increased demand for livestock products, especially meat production, the problem of livestock feed shortage is particularly prominent. Thus, resource exploitation is imperative strategy to reduce the use of conventional feed in animal production. Okara, a byproduct from the soymilk and tofu industry, was produced annually about 2.8 megatons in China and around 14 megatons worldwide and was a major agro‐waste due to huge quantities of the abandoned okara (Choi, Kim, Jung, & Bae, 2015; Li et al., 2013). Okara contains a high nutritive value with 50% crude fiber, 25% protein, 10% to 15% oil as well as a myriad of bio‐active substance such as isoflavones, polysaccharides, coumestans, saponins, phytosterols, and phytates (Mok, Tan, Lee, Kim, & Chen, 2019; O'Toole, 1999), its reuse as a feed resources for animals is an economical and environmental friendly option (Gupta, Lee, & Chen, 2018). However, okara has been primarily disposed of in landfills and by incineration due to its unpalatable and insoluble nature. Thus, it is necessary to release the nutrients from the insoluble okara and improve its palatability before it can be applied in the pig industry.

The fermentation process can change the physicochemical properties and improve the nutritional value (Li, Chen, Li, Lei, & Zhang, 2016) such as making the essential and nonessential amino acids of okara available (Frias, Song, Martinez‐Villaluenga, Gonzalez, & Vidal‐Valverde, 2008; Shi, Yang, Guan, Wang, & Zhang, 2013; Wang, Liu, Wang, Cheng, & Mou, 2014). Zhou et al. (2019) reported that fermented okara had a prebiotic function and could decrease intestinal pH value and ammonia N concentration and increase short‐chain fatty acids (SCFA). Furthermore, antinutritional factors and other hazardous compounds of okara were detoxified by fermentation (Mukherjee, Chakraborty, & Dutta, 2016), which is possible due to microbial fermentation can also provide useful metabolites such as antibiotic peptides, prebiotics, and digestive enzymes (Lio & Wang, 2012; Mok et al., 2019; Seo & Lee, 2004), thereby enhancing immunity, digestive, and absorptive function in animals.

Despite the potential of gaining access to a massive feed resource, there has been relatively little study to evaluate the efficacy of probiotics fermented okara on growth performance and meat quality in pigs. Therefore, the objective of this study was to investigate characteristics of fermented okara (FO) and its efficacy on growth performance and meat quality in growing pigs, thereby exploring the feasibility of conventional feed substitution with FO in the pig industry.

## 2 MATERIALS AND METHODS

### 2.1 Fermented okara

Components of diets are shown in Table 1. Fermented okara was prepared using commercial probiotics including *Saccharomyces cerevisiae*, *Bacillus subtilis,* and *Lactobacillus plantarum* (purchased from Guangdong Microbial Culture Collection Center) according to a previously optimized fermentation method. Briefly, okara with 60% to 70% moisture was inoculated with 1 × 10^9^ CFU/kg of a complex probiotics of *S. cerevisiae*, *B. subtilis,* and *L. plantarum* in the proportions of 1:1:1. The anaerobic fermentation was processed at 35°C for 8 d after a thorough mixing. At end of the fermentation, samples were collected and stored at −80°C until further analysis. FO was used alongside unfermented okara (UFO) in the following experiments and analysis.

**Table 1:** Formulation and chemical composition of the experimental diets (as‐fed basis))

Ingredient (%)	Control	FO
Corn	66	36.6
Soybean meal	25	18
Wheat bran	8	0
FSCR	0	36.6
Wheat hull	0	7.8
Premix^a^	1	1
Total	100	100
Nutrition level (%)^b^		
Digestible energy, Kcal/kg	3,228	3,328
Crude protein	16.54	15.96
Calcium	0.58	0.23
Total P	0.55	0.53
Available P	0.23	0.25
Lysine	0.74	1.01
Methionine	0.27	0.26
Methionine + Cysteine	0.56	0.55
Threnine	0.56	0.54
Tryptophane	0.16	0.18

^a^ Premix provided these amounts of vitamins and minerals per kilogram on an as‐fed basis for growing pigs: 1, 750 IU/kg vitamin A, 220 IU/kg vitamin D3, 3 IU/kg vitamin E, 0.55 mg/kg vitamin K3, 0.25 mg/kg vitamin B1, 1.0 mg/kg vitamin B2, 0.7 mg/kg vitamin B6, 3 µg/kg vitamin B12, 4 mg/kg niacin, 1.6 mg/kg calcium pantothenate, 0.1 mg/kg folic acid, 7 µg/kg biotic, 0.08 g/kg choline chloride, 6.5 mg/kg manganese, 15 mg/kg iron, 15 mg/kg zinc, 1.5 mg/kg copper, 0.07 mg/kg iodine (I_2_), 0.03 mg/kg selenium, and 1 g/kg sodium chloride.

^b^ The values are expressed as percentage (%), except for digestible energy.

### 2.2 Nutrient compositive analysis of fermented okara

Determination of protein content, fat, and moisture was conducted using methods described in the National Standards of People's Republic of China (GB/T 5009.5–2003). Polysaccharide contents of UFO and FO were determined using the phenol‐sulfuric acid method. Samples were taken before and after fermentation for electron microscopy and prepared with the ion sputter coating method before being placed in the scanning electron microscope sample chamber (Hitachi S3700 Scanning Electron Microscope), the voltage was set to 5 kV and samples photographed at 500 times and 2, 000 times magnification to observe the morphology of the material's surface.

### 2.3 Experimental design, animals, and diets

All procedures for the animal experiments were approved by the Animal Care and Use Committee of Guangdong Academy of Agricultural Sciences.

A total of 48 pigs (Duroc × Landrace × Yorkshire) with an average body weight of 58.60 ± 0.65 kg were randomly assigned to 2 groups, Control group and FO group. Each group comprised 8 replicate pens with 3 pigs per pen. Control pigs were fed a corn‐soybean meal basal diet, treatment pigs were fed a basal diet supplemented with FO (Table 1). All pigs were housed in cages and had ad libitum access to water and feed throughout the 55‐d experimental period.

### 2.4 Growth performance

Average feed intake per group was calculated weekly according to daily feed intake per pen. All pigs were weighed at the beginning and end of animal experiment to evaluate growth performance, and the following parameters were calculated: average daily gain (ADG), average daily feed intake (ADFI), and the feed to gain ratio (F/G) at the end of the experiment.

### 2.5 Slaughter procedure and sample collection

At the end of the experiment, all pigs were fasted for 12 hr and the pig closest to the average weight per pen was selected and blooded from the ear marginal veins using heparin‐coated vacutainer tubes. Blood was centrifuged for 15 min at 3, 500 g at 4°C and the supernatant was separated into 4 plastic vials and stored at −80°C. Pigs were sacrificed after electro‐stunning, followed by exsanguination and the collection of muscle samples. About 6 g of the left *longissimus thoracis* (LT), *biceps femoris* (BF), and s*emitendinosus* (ST) muscles were immediately collected and placed into six sterile Eppendorf tubes, and another 50‐g of each muscle was subpackaged in two bags, frozen in liquid nitrogen, and then stored at −80°C until measurement for biochemical parameters, antioxidant capability, free amino acids (FAA), and intramuscular fat (IMF).

### 2.6 Meat quality

According to the standards of the National Pork Producers Council (NPPC, 1991), pH value and meat color CIE LAB value (L*, a*, and b*) of the *longissimus thoracis* (LT) were determined on the 10th rib using a portable pH meter equipped with glass electrode (testo‐205, Testo, Germany) and a colorimeter (CR‐410, Minolta, Japan) at 45 min, and then they were detected again after refrigerating at 4 ºC for 24 hr and 48 hr postslaughter. The marbling scores were determined subjectively (from 0 to 4, where 0 = devoid and 4 = overly abundant), and the average marbling score for each sample was calculated from multiple observers. Drip loss of LT muscle was measured and indirectly reflected water‐holding capacity (Mason et al., 2005). Muscle cores of 25 mm diameter (six cores per pig) were collected by a cork borer and refrigerated at 4°C after weighing, hooking, and wrapping in plastic bags. The three LM samples were weighed after removing its surface water for 24 hr and 48 hr. Drip loss was calculated according to initial and final weight of LT muscles. Shear force was determined as follows: After refrigerating at 4ºC for 24 hr, LT muscle (approximately 250 g per pig) was cooked in 80ºC water until reaching 70ºC inside and cooled to 25ºC. Cylindrical pork chops (10‐mm diameter × 10‐mm length) were cut perpendicularly to the muscular fiber orientation by an Instron Universal Mechanical Machine (Instron model 4,411, Instron Corp., Canton) equipped with a Warner‐Bratzler type of blade at a cross‐head speed of 127 mm/min. The average values were calculated and expressed in Newtons as shear force per sample.

The IMF content was determined using the Soxhlet method (Luque De Castro & Priego‐Capote, 2010). Fresh muscle samples (approximately 30 g) were gradually smashed, lyophilized, and pulverized. Lipids were extracted from homogenized powder (3 g) using petroleum ether (AOAOC, 2000) and then analyzed using the Soxtec 2055 fat extraction system (Foss Tecator AB, Foss, Sweden).

### 2.7 Free amino acids

FAA were measured in serum, LT muscle (Table 7), BF, and ST muscles (Table S1) of growing pigs. Approximately 1 g muscle sample was homogenized and de‐proteinized with 3 ml sulfosalicylic acid (20% W/V), and then centrifuged for 15 min at 12,000 *g* at 4°C. Supernatants were filtered using a 0.22 μm filter and then analyzed for FAA using an automatic amino acid L‐8900 analyzer (Hitachi, Tokyo, Japan).

### 2.8 Biochemical parameters and antioxidant capability analysis

Muscle was homogenized in 0.9% NaCl at the proportion of 1:3 (W/V) and centrifuged for 15 min at 3, 500 g at 4°C and the supernatant was collected. Following the protocols provided with the analysis kits (Nanjing Jiancheng, Nanjing, China), the content of oxidative product MDA (malondialdehyde) and activities of antioxidant enzymes like T‐SOD (total superoxide dismutase), GSH‐PX (glutathione peroxidase), and CAT (catalase) were determined in the muscular supernatant and plasma of growing pigs. Plasmatic cholesterol, triglyceride (TG), glucose, and muscle glycogen were also determined using the appropriate kits (Nanjing Jiancheng, Nanjing, China).

### 2.9 Statistical analysis

All experimental data were analyzed using GraphPad Prism 6 (GraphPad software, Inc. San Diego, CA, USA) and processed by one‐way analysis of variance (ANOVA) with Tukey's post hoc test. Statistical significance was set at *p* ≤ .05 and statistically significant trends at *p* ≤ .10.

## 3 RESULTS

### 3.1 Characteristic of fermented and unfermented okara

**Figure 1:**
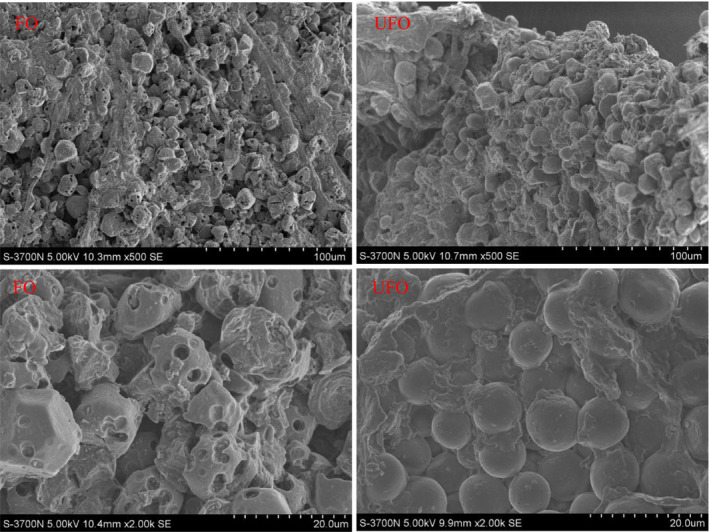
Morphologic structures of unfermented and fermented okara. Morphologic structures of fermented (Left) and unfermented okara (Right) by scanning electron microscope (bar: 100 µm and 20 µm). FO, fermentated okara; UFO, unfermented okara

After fermentation for 8 d, the external sensory quality of okara was improved with no agglomeration accompanied by an acidic fragrance. The morphological observation of UFO and FO using scanning electron microscopy suggested that FO had a more microporous structure and was looser than UFO (Figure 1). The compositions of UFO and FO are shown in Table 2. Compared to UFO, FO had higher content of polysaccharide, lactic acid, and energy, but lower content of crude protein, moisture, and crude fat.

**Table 2:** Composition of okara before and after fermentation with probiotics

Items	UFO	FO	*SEM*	*P*‐Value
Moisture content (%)	77.85^a^	64.61^b^	0.12	<.01
Crude protein (%)	17.69^a^	15.80^b^	0.18	<.01
Crude fat (%)	1.09^a^	0.66^b^	0.03	<.001
Polysaccharide (mg/g)	354.89^b^	518.35^a^	8.23	<.001
Lactic acid (μmol/g)	83.46^b^	208.29^a^	5.06	<.001
Energy (MJ/kg)	18.03^b^	18.90^a^	0.06	<.01

^a,b^Values in a row with different superscripts differ significantly (*p* ≤ .05).

Abbreviations: FO, fermented okara; *SEM*, standard error of the mean; UFO, unfermented okara.

After fermentation, the content of total FAA of FO was higher than UFO (Table 3, *p* < .01). Apart from lysine and histidine, the contents of all other amino acids increased significantly in FO compared with UFO (*p* < .05).

**Table 3:** Free amino acid composition of okara before and after fermentation with probiotics (mg/100 g)

Composition (%)	UFO	FO	*SEM*	*P*‐value
Asparagic acid	0.49^b^	0.68^a^	0.02	<.01
Threnine	0.22^b^	0.33^a^	0.07	<.01
Serine	0.28^b^	0.40^a^	0.10	<.01
Glutamic aicd	0.68^b^	1.17^a^	0.01	<.01
Glycine	0.14^b^	0.20^a^	0.0	<.01
Alanine	0.28^b^	0.55^a^	0.0	<.01
Valine	0.25^b^	0.34^a^	0.03	.02
Cysteine	2.31^b^	3.32^a^	0.24	.05
Isoleucine	0.11^b^	0.22^a^	0.01	<.01
Leucine	0.35^b^	0.68^a^	0.24	<.01
Tyrosine	0.16^b^	0.30^a^	0.02	<.01
Phenylalanine	0.25^b^	0.39^a^	0.02	<.01
Lysine	0.29	0.30	0.01	.46
Histidine	0.38	0.41	0.42	.95
Arginine	0.44^b^	0.51^a^	0.03	.05
Proline	0.32^b^	0.52^a^	0.04	.01
Total free amino acids	6.26^b^	10.42^a^	0.37	<.01

^a,b^Values in a row with different superscripts differ significantly (*p* ≤ .05).

Abbreviations: FO, fermented okara; *SEM*, standard error of the mean; UFO, unfermented okara.

### 3.2 Growth performance

Growth performance of growing pigs is shown in Table 4, pigs fed with FO had higher ADG and lower F/G than pigs fed the control diet (*p* < .05). There were no significant differences in FBW and ADFI between the two groups (*p* > .05).

**Table 4:** Effect of diet supplemented with fermented okara (FO) on growth performance of growing pigs

Item	Control	FO	*SEM*	*P*‐Value
IBW (kg)	59.19	58.00	0.65	.09
FBW (kg)	97.16	99.09	0.19	.13
ADG (kg/d)	0.69^b^	0.75^a^	0.02	<.01
ADFI (kg)	2.11	2.17	0.05	.23
F/G	3.06^a^	2.89^ b^	0.08	.04

^a,b^Values in a row with different superscripts differ significantly (*p* ≤ .05).

Abbreviations: ADFI, average daily feed intake, ADG, average daily gain, F/G, feed to gain ratio, FBW, final body weight, FO, fermented okara, IBW, initial body weight, *SEM*, standard error of the mean.

### 3.3 Biochemical parameters and antioxidant capability

As shown in Table 5, no differences were observed in plasmatic biochemical parameters such as TG, cholesterol, glucose, and muscle glycogen in plasma or LT muscle between the control diet and the diet supplemented with FO (*p* > .05). Diet supplemented with FO significantly increased activities of antioxidant enzymes like T‐SOD and GSH‐Px (*p* < .05), but had no effect on oxidative product MDA or CAT activity in plasma or the LT muscle of pigs in comparison with the control diet (*p* > .05).

**Table 5:** Effect of diet supplemented with fermented okara (FO) on biochemical parameters and antioxidant capability in serum and *longissimus thoracis* muscle of pigs

Items	Control	FO	*SEM*	*P*‐Value
Serum				
TG, mmol/L	0.44	0.39	0.03	0.11
Cholesterol, mmol/L	2.01	2.13	0.17	0.17
Glucose, mmol/L	4.78	4.96	0.13	0.17
MDA, nmol/L	1.67	1.72	0.05	0.73
T‐SOD, U/mL	58.86^b^	62.44^a^	1.30	0.01
GSH‐Px, U	777.21^b^	841.69^a^	27.06	0.02
CAT, U/mL	4.36	4.73	0.45	0.16
Muscle				
Glycogen, mg/g	2.59	2.71	0.32	0.72
MDA, nmol/mg	0.15	0.15	0.01	0.86
T‐SOD, U/mg	4.58^b^	4.96^a^	0.15	0.01
GSH‐Px, mg/g	3.85^b^	5.37^a^	0.40	< 0.01

^a,b^Values in a row with different superscripts differ significantly (*p* ≤ .05).

Abbreviations: CAT, catalase; FO, fermented okara; GSH‐Px, gluathione peroxidase; MDA, malondialdehyde; *SEM*, standard error of the mean; TG, triglyceride; T‐SOD, total superoxide dismutase.

### 3.4 Meat quality

Meat quality of LT muscle is shown in Table 6. No significant differences were observed in pH, drop loss, marbling score, or shear force between the two groups (*p* > .05). The LT muscles of pigs fed FO had higher the a^*^ value at 48 hr (*p* < .05), tended to have lower the b^*^ value at 45 min and 48 hr, but the difference was no statistically significant (*p* > .05) than pigs fed the control diet.

**Table 6:** Effect of diet supplemented with fermented okara (FO) on meat quality of *longissimus thoracis* muscle in growing pigs

Items	Control	FO	*SEM*	*P*‐Value
pH_45 min_	6.62	6.61	0.10	.962
pH_24 hr_	5.81	5.73	0.06	.614
pH_48 hr_	5.55	5.60	0.03	.213
L*_45 min_	47.04	46.28	0.71	.310
a*_45 min_	16.97	16.74	0.32	.491
b*_45 min_	2.86	2.13	0.29	.098
L*_24 hr_	50.36	51.88	1.52	.342
a*_24 hr_	16.45	16.77	0.45	.493
b*_24 hr_	3.15	2.91	0.43	.391
L*_48 hr_	56.17	54.96	0.98	.244
a*_48 hr_	16.75^b^	17.5^a^	0.32	.011
b*_48 hr_	3.35	2.91	0.24	.067
Drop loss (%)_24 hr_	1.81	1.88	0.07	.301
Drop loss (%)_48 hr_	1.60	1.51	0.04	.513
Marbling score	3.01	2.85	0.33	.552
Shear force (*N*)	52.45	53.50	2.64	.701

^a,b^Values in a row with different superscripts differ significantly (*p* ≤ .05).

Abbreviations: FO: fermented okara; *SEM*, standard error of the mean.

### 3.5 Intramuscular fat

**Figure 2:**
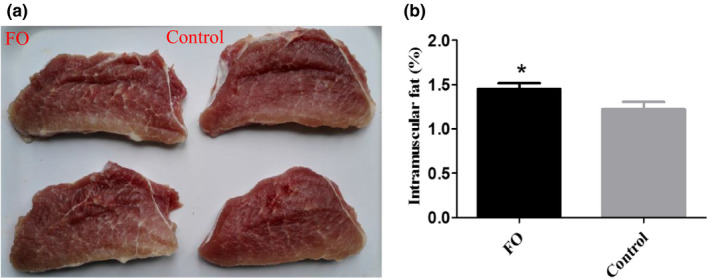
Intramuscular fat of the *longissimus thoracis* (LT) muscle in growing pigs. (a) Observation of intamuscular fat by image of LT muscle using digital camera (Canon, Tokyo, Japan). (b) The content of intramuscular fat was determined according to the Soxhlet method. All values are expressed as mean ± *SEM* (*n* = 8). *means as statistically significant at *p* ≤ .05 from applying one‐way ANOVA followed by Tukey's post hoc test. Control, a basal diet; FO, fermentated okara

Intramuscular fat content of pork was detected in growing pigs (in Figure 2 and Figure S1). More intramuscular fat was observed in LT muscle of pigs fed the diet supplemented FO than pigs fed the control diet (Figure 2a,b). Furthermore, intramuscular fat content was prominently enhanced in BF and ST (Figure S1) muscles of pigs fed the diet supplemented with FO compared to pigs fed the control diet (*p* < .05).

### 3.6 Amino acids profiles

As shown in Table 7, compared to the control diet, the diet supplemented with FO significantly increased threonine and proline in the LT, and glutamine in serum (*p* < .05), and tended to increase levels of threonine, alanine and tyrosine in serum (0.05 < *p* ≤ .10). As shown in Table S1, levels of threonine in BF muscle and levels of threonine, serine, and asparagine in ST muscle were higher (*p* ≤ .05), whereas asparagine level in ST muscle was lower in pigs fed the diet supplemented with FO than pigs fed the control diet (*p* = .05).

**Table 7:** Effect of diet supplemented with FO on free amino acids profile in serum (nmol/μL) and *longissimus thoracis* muscle (mg/100 g)

Items	Serum		*SEM*	*P* value	*longissimus thoracis*		*SEM*	*P* value
	FO	Control			FO^2^	Control		
Histidine	0.07	0.07	0.00	.75	1.96	1.89	0.03	.70
Isoleucine	0.08	0.08	0.00	.83	2.22	2.10	0.06	.47
Leucine	0.15	0.16	0.01	.70	3.54	3.34	0.10	.73
Lysine	0.17	0.16	0.01	.75	4.45	4.28	0.09	.82
Methionine	0.03	0.03	0.00	.34	2.19	2.37	0.09	.86
Phenylalanine	0.09	0.10	0.00	.27	3.00	3.04	0.02	.85
Threnine	0.15	0.12	0.01	.08	4.05^a^	3.06^b^	0.50	.01
Valine	0.22	0.23	0.01	.67	4.48	4.46	0.01	.98
Alanine	0.42	0.35	0.04	.08	26.51	24.36	1.08	.43
Arginine	0.11	0.09	0.01	.26	3.13	2.92	0.11	.67
Asparagine	0.03	0.04	0.00	.89	1.60	1.42	0.09	.36
Glutamic acid	0.07	0.07	0.00	.61	3.54	3.42	0.06	.83
Glutamine	0.41^a^	0.33^b^	0.04	.01	38.47	41.22	1.38	.72
Glycine	0.59	0.52	0.03	.49	11.16	10.51	0.33	.45
Proline	0.17	0.14	0.01	.22	4.46^a^	3.14^b^	0.66	< .01
Serine	0.09	0.08	0.00	.52	2.93	2.51	0.21	.11
Tyrosine	0.09	0.07	0.01	.07	3.58	3.22	0.18	.26
Cysteine	0.03	0.03	0.00	.86	0.56	1.25	0.35	.55
Asparagic acid	0.01	0.01	0.00	.72	0.44	0.61	0.09	.32
NEAA	2.02	1.72	0.16	.38	95.67	93.97	0.85	.88
EAA	0.96	0.95	0.15	.19	21.41	20.09	0.66	.65
Tasty AA	1.09	0.95	0.00	.99	42.81	39.71	1.55	.37
Total AA	2.98	2.67	0.15	.41	122.00	119.00	1.44	.82

^a,b^Values in a row with different superscripts differ significantly(*p* < .05) and *P*‐value ≤ 0.10 was considered as tendency toward statistical significance.

Abbreviations: EAA: essential AA, including histidine, isoleucine, leucine, lysine, methionine, phenylalanine, threnin, and valine; FO, fermented okara; NEAA, nonessential AA, including alanine, arginine, asparagine, glutamic acid, glutamine, glycine, proline, serine, tyrosine, and cysteine; *SEM*, standard error of the mean; Tasty AA includes alanine, aspartic acid, glutamic acid, and glycine.

## 4 DISCUSSION

Okara is produced from soy products of soymilk and bean curd and is generally discarded, which results in the waste of an untapped resource and environment pollution. The data in the present study revealed that okara contained 17.69% crude protein and had a high potential value as a low‐cost plant protein resource for the pig industry. Okara contains antitrypsin, saponin, hemagglutinin, and other antinutritional factors such as trypsin inhibitor, thereby inhibited the digestion and absorption of nutrients in pigs and limited its practical applications (Li et al., 2013). In the present study, okara had a more microporous structure and was looser by probiotics fermentation, which likely promotes digestion and absorption of okara.

Waliszewski, Pardio, and Carreon (2002) also pointed out that okara had a ratio of essential amino acids (EAA) to total amino acids similar to those of soy milk and bean curd. The present results displayed that probiotics fermentation improves 16 kinds of FAA and total FAA content, suggesting the fermentation of okara can improve its nutrient values of amino acids. The result accorded with Rashad, Mahmoud, Abdou, and Nooman (2011) that probiotics fermentation can enhance nutrient quality of okara. However, due to high moisture, unpalatable nature, and off‐flavor undesirable flavor, okara was rarely applied in pig breeding. Previous studies reported that probiotics can biotransform volatiles such as acids, aldehydes, alcohols, and eaters, thereby reducing or masking off‐odors (Ghosh et al., 2015; Sumby et al., 2010), and our results agreed with reports that FO had lower moisture, a perceptibly acidic and fruity fragrance than UFO. Furthermore, FO had higher concentrations of lactic acid and polysaccharides, which accorded with previous studies that microbial solid‐state fermentation of okara decreased carbohydrate metabolism and increased glycolysis in okara, and subsequently utilized the glycolysis intermediate to produce polysaccharides, organic acid concentrations, and amino acids (Frias et al., 2008; Giang, Viet, Ogle, & Lindberg, 2012; Li et al., 2016; Mok et al., 2019). It also explains why okara had lower crude protein, crude fat after fermentation. Consequently, these alterations improved palatability, flavor, and the nutrient value of the feed (Quintana, Gerbino, & Gómez‐Zavaglia, 2017).

Rashad et al. (2011) reported that nutrient quality of okara was enhanced through fermentation using yeast strains. Previous researches revealed that probiotics *B. subtilis* and *L. plantarum* improved growth performance and carcass quality in pigs (Alexopoulos et al., 2004; Suo et al., 2012), and fermented okara had enhanced cellulases, xylanases, and nutrient profile by *B. subtilis* (Heck, Hertz, & Ayub, 2002; Mok et al., 2019) and had improved unsaturated/saturated (U/S) fatty acid ratio by *L. plantarum* (Quintana et al., 2017). Therefore, in the current study, fermentation of okara was processed and optimized using commercial compound probiotics including *S. cerevisiae*, *B. subtilis,* and *L. plantarum*, which finally obtained an effective FO. In the present study, FO was used to replace 44.55% corn, 28% soybean meal, and 100% wheat bran of corn‐soybean meal‐wheat bran diets, respectively. Interestingly, FO significantly improved ADG and reduced F/G, but did not significantly increase FBW of growing pigs, suggesting that FO can substitute partly corn‐soybean meal‐wheat bran for pig production. Previous reports had discovered that FO strengthened antioxidant activity, thereby displaying the free radical scavenging ability (Mok et al., 2019; Zhu, Fan, Cheng, & Li, 2008), which agreed with our study in that the diet supplemented with FO promoted antioxidant capacity by increasing T‐SOD and GSH‐Px activities in the blood and muscle of growing pigs. This may because okara increased polysaccharides to enhance itself antioxidant activities through probiotics fermentation (Rashad et al., 2011). In addition, polysaccharides are conducive to enhancing the antioxidant capacity of animals as physiological active matter (Mukherjee et al., 2016; Shi, Yang, Hu, & Zhang, 2014; Vong, Au, & Liu, 2016; Vong & Liu, 2018), except for its the immunomodulatory functions and antioxidant properties (Ooi & Liu, 2000; Yuan, Zhang, Fan, & Yang, 2008). Probiotics utilized carbohydrates of okara to produce energy and other components through glycolysis pathway, thereby promoting its metabolic, cellular processes, and antioxidant capacity (Mok et al., 2019), which agreed to the result in this study that probiotics fermented increased okara energy. Additionally, probiotics also had a growth‐promoting function (Alexopoulos et al., 2004; Jiang et al., 2015), ameliorated nutrition digestibility, and balanced intestinal microflora (Gupta et al., 2018; Lan, Lee, & Kim, 2016; Yang, Jiang, Zheng, Wang, & Yang, 2014). Collectively, the growth‐promoting function of FO is likely due to increased polysaccharide abundance, prebiotic function of probiotics, and induced antioxidant capacity.

Meat quality is the decisive factor for consumers to make the purchasing decisions, especially meat color. Because meat color is the key indicator of discoloration, freshness, and wholesomeness than any other quality factor in the sight of consumers. Shidara et al. (2005) reported that dry okara significantly reduced meat quality, especially the juiciness and the CIE a* value of pork. However, published research is little known about a role for FO in regulating pork quality. In this study, the diet supplemented with FO decreased b* at 45 min and 48 hr, and increased a* at 48 hr, suggesting a regulatory role of FO on meat quality in pigs. These results likely because *S. cerevisiae*, *B. subtilis,* and *L. plantarum* had prebiotic effects on improving pork quality such as pH, hardness, stickiness, meat color, and fat content (Baowei, Guoqing, Qiaoli, & Bin, 2011; Kovacs‐Zomborszky, Feher, & Soos, 1997; Sheng et al., 2016; Suo et al., 2012).Therefore, FO seems to serve as a functional feed to improve pork quality by regulating meat color such as b* and a* values, especially at 48 hr postmortem. Previous research declared that a* values were correlated to pigment content and redox state, but b* was only related to redox state rather than pigment content (Lindahl, Lundstrom, & Tornberg, 2001; Mancini & Hunt, 2005), which accorded to results in this study. The present results displayed that diet supplemented with FO increased antioxidant capacity by inducing T‐SOD and GSH‐Px in serum and muscle, increased a* values, but decreased b* values. In addition, polysaccharides have been shown to improve meat quality by regulating meat color and antioxidant capacity (Ma et al., 2017), whereas antioxidant capacity of muscle is closely related to meat quality (Jiang & Xiong, 2016; Ma et al., 2010; Zhang et al., 2015). Therefore, we infer that FO improve meat quality by increasing polysaccharides to improve meat color and antioxidant capacity of pork.

Intramuscular fat content of pork is tightly correlated with meat quality, especially eating quality (Jeong et al., 2010), and is positively related to juiciness, flavor, and tenderness (Alonso, Campo, Provincial, Roncales, & Beltran, 2010; Wood et al., 2008). Our results suggested that intramuscular fat was improved in pigs fed dietary FO compared with pigs fed the control diet, suggesting that dietary FO improve eating quality of pork. Furthermore, polysaccharides have been shown to improve meat quality by regulating intramuscular fat deposition (Ma et al., 2017). Therefore, increased polysaccharides in FO likely improved intramuscular fat, thereby improving eating quality of pork.

Muscular FAA are mainly categorized into three kinds, fresh, sweet, and bitter, according to their flavor. Generally, fresh amino acids include glycine, proline, serine, and alanine; sweet amino acids include proline, alanine, glycine, serine, threonine, lysine, cysteine, asparagine and glutamine, and bitter amino acids include methionine, valine, leucine, isoleucine, phenylalanine, and tyrosine (Kato, Rhue, & Nishimura, 1989; Kikkawa, Toko, Matsuno, & Yamafuji, 1993; Lorenzo & Franco, 2012). Muscular FAA are directly related to the nutrient value and flavor as taste enhancers or precursors of aroma compounds in pork (Kato et al., 1989; Li et al., 2018). Previous research had pointed out that fermentation of okara decreased carbohydrate metabolites, but increased amino acids production and altered amino acids composition by upregulating glycolysis (Mok et al., 2019), clarifying the reason why diet with FO increased plasma and muscular amino acids profile. It is noteworthy that higher contents of sweet and fresh amino acids such as muscular free threonine and proline, serum alanine, glycine, and serine were observed in pigs fed diet with FO, even lower alanine of BF muscle and glutamine of SF muscle were observed in pigs fed diet with FO.

Previous studies suggested that the abundances of amino acids and fatty acids profiles of okara were enhanced by fermentation using probiotics included *S. cerevisiae*, *B. subtilis,* and *L. plantarum*, thereby increasing fat deposition and amino acids nutrient value of pork (Gupta et al., 2018; Mok et al., 2019; Vong et al., 2016), which explains why diet supplemented with FO can increased fat and amino acid content of pork in present study. Taken together, FO ameliorated pork quality by improving intramuscular fat and flavor amino acids in pigs.

## 5 CONCLUSION

In brief, the fermentation of okara using *S. cerevisiae, B. licheniformis,* and *L. plantarum* accelerate its microporous structure and acid fragrance by increasing lactic acid and polysaccharides. Furthermore, FO can substitute the partial corn‐soybean‐wheat bran to promote growth by increasing ADG, improve antioxidant capacity by increasing SOD and GSH‐Px, and ameliorate meat quality by increasing a* values and intramuscular fat and decreasing b* values of pork. This study also provided an innovative strategy for economic, environmentally friendly and resource exploitation and utilization of okara as a functional feed for pig production.

## CONFLICT OF INTEREST

The authors declare no conflict of interest.

## ETHICAL APPROVAL

Animal procedures experiments were approved by the Animal Care and Use Committee of Guangdong Academy of Agricultural Sciences.

## Supporting information

Supplementary Material
