# Supplementary material for: Diet supplemented with fermented okara improved growth performance, meat quality, and amino acid profiles in growing pigs
Source: Food Sci Nutr. 2020 Sep 7;8(10):5650–9. doi: 10.1002/fsn3.1857 (PMC7590273; doi:10.1002/fsn3.1857)
Supplement: Supplementary file 1 — Supplementary Material [file FSN3-8-5650-s001.docx]

**Supplementary material**

**Diet supplemented with fermented okara improved growth performance, meat quality and amino acid profiles in growing pigs**

Running title: Fermented okara improved growth and meat quality

Zhimei Tian^#^, Dun Deng^#^, Yiyan Cui, Weidong Chen, Miao Yu, Xianyong Ma^[[1]](#footnote-1)^*

State Key Laboratory of Livestock and Poultry Breeding, Key Laboratory of Animal Nutrition and Feed Science in South China, Ministry of Agriculture and Rural Affairs, Guangdong Key Laboratory of Animal Breeding and Nutrition, Guangdong Engineering Technology Research Center of animal Meat quality and Safety Control and Evaluation, Institute of Animal Science, Guangdong Academy of Agricultural Sciences, 1 Dafeng 1st street, Wushan road, Tianhe district, Guangzhou 510640, China;

* Correspondence: Tel.: +86 020-861368896; fax: +86 020-861368896.

E-mail address: [maxianyong@gdaas.cn](mailto:maxianyong@gdaas.cn) (X. Ma)

**Supplementary material**

**Summary**

The supporting information includes one table and one figure.

**Table S1.** Effect of diet supplemented with FO on free amino acids profile in *biceps femoris and semitendinosus* muscles (mg/100 g)

| Items^1^ | *Biceps femoris* muscle | | SEM^3^ | *P* Value | *Semitendinosus* muscle | | SEM^3^ | *P* Value |
| --- | --- | --- | --- | --- | --- | --- | --- | --- |
|  | FO^2^ | Control |  |  | FO^2^ | Control |  |  |
| Histidine | 2.66 | 2.72 | 0.03 | 0.84 | 2.72 | 2.97 | 0.13 | 0.41 |
| Isoleucine | 2.30 | 2.22 | 0.04 | 0.68 | 2.26 | 2.24 | 0.01 | 0.94 |
| Leucine | 4.16 | 4.23 | 0.04 | 0.90 | 3.97 | 4.13 | 0.08 | 0.71 |
| Lysine | 5.78 | 5.80 | 0.01 | 0.99 | 5.80 | 6.59 | 0.40 | 0.42 |
| Methionine | 2.12 | 2.18 | 0.03 | 0.72 | 1.60 | 1.50 | 0.05 | 0.60 |
| Phenylalanine | 3.62 | 3.75 | 0.07 | 0.60 | 3.19 | 3.40 | 0.11 | 0.33 |
| Threnine | 4.70^a^ | 3.76^b^ | 0.47 | 0.01 | 6.01^a^ | 4.67^b^ | 0.67 | 0.04 |
| Valine | 4.41 | 4.54 | 0.07 | 0.80 | 4.80 | 4.56 | 0.12 | 0.80 |
| Alanine | 36.80 | 37.14 | 0.17 | 0.91 | 42.09 | 40.30 | 0.90 | 0.69 |
| Arginine | 3.73 | 3.62 | 0.05 | 0.83 | 3.88 | 4.39 | 0.26 | 0.70 |
| Asparagine | 2.03 | 2.08 | 0.03 | 0.82 | 2.36^b^ | 2.50^a^ | 0.07 | 0.05 |
| Glutamic acid | 4.09 | 4.36 | 0.14 | 0.77 | 5.86 | 8.45 | 1.29 | 0.54 |
| Glutamine | 50.66 | 49.02 | 0.82 | 0.80 | 56.36 | 62.95 | 3.29 | 0.14 |
| Glycine | 13.41 | 14.19 | 0.39 | 0.57 | 16.34 | 16.31 | 0.02 | 0.67 |
| Proline | 3.90 | 3.42 | 0.24 | 0.28 | 6.32 | 4.28 | 1.02 | 0.98 |
| Serine | 3.90 | 3.67 | 0.11 | 0.54 | 4.60^a^ | 4.24^b^ | 0.18 | 0.02 |
| Tyrosine | 3.89 | 3.59 | 0.15 | 0.14 | 3.71 | 3.44 | 0.13 | 0.48 |
| Cysteine | 0.48 | 0.48 | 0.00 | 0.99 | 0.37 | 0.21 | 0.08 | 0.23 |
| Asparagic acid | 1.77 | 1.16 | 0.31 | 0.19 | 1.38 | 1.19 | 0.09 | 0.77 |
| NEAA | 122.90 | 121.58 | 0.66 | 0.91 | 141.90 | 147.07 | 2.59 | 0.82 |
| EAA | 25.34 | 24.66 | 0.34 | 0.75 | 25.55 | 25.50 | 0.02 | 0.98 |
| Tasty AA | 56.33 | 57.78 | 0.72 | 0.75 | 66.65 | 67.56 | 0.45 | 0.89 |
| Total AA | 154.42 | 151.94 | 1.24 | 0.85 | 173.62 | 178.33 | 2.35 | 0.84 |

^a,b^Values in a row with different superscripts differ significantly(*P* < 0.05) and *P*-value ≤ 0.10 was considered as tendency toward statistical significance.

^1^ NEAA: non-essential AA, including alanine, arginine, asparagine, glutamic acid, glutamine, glycine, proline, serine, tyrosine and cysteine; EAA: essential AA, including histidine, isoleucine, leucine, lysine, methionine, phenylalanine, threnin and valine; Tasty AA includes alanine, aspartic acid, glutamic acid and glycine. ^2^ FO: fermented okara; ^3^SEM means standard error of the mean.


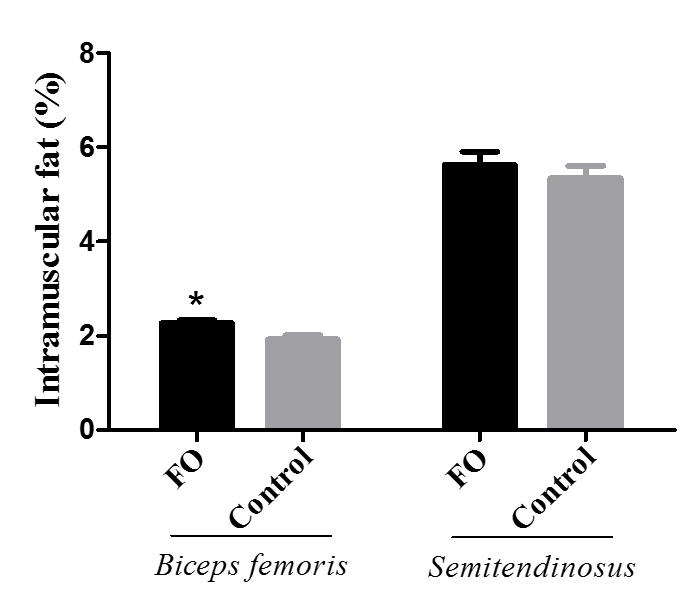


**FIGURE S1** Intramuscular fat content of *biceps femoris* (BF) and semitendinosus (ST) muscles in growing pigs

Intramuscular fat content of was determined in BF and ST muscles according to the Soxhlet method. All values are expressed as mean ± SEM (n=8). *means as statistically significant at P ≤ 0.05 from applying one-way ANOVA followed by Tukey’s post hoc test. Control, a basal diet; FO, fermentated okara.

1. *Corresponding author: Xianyong Ma. E-mail: [xianyongm@gmail.com](mailto:xianyongm@gmail.com), Tel: (+86) 020-61368896

   ^#^These authors contributed equally to this work [↑](#footnote-ref-1)
